# Daratumumab in transplant-eligible patients with newly diagnosed multiple myeloma: final analysis of clinically relevant subgroups in GRIFFIN

**DOI:** 10.1038/s41408-024-01088-6

**Published:** 2024-07-08

**Authors:** Ajai Chari, Jonathan L. Kaufman, Jacob Laubach, Douglas W. Sborov, Brandi Reeves, Cesar Rodriguez, Rebecca Silbermann, Luciano J. Costa, Larry D. Anderson, Nitya Nathwani, Nina Shah, Naresh Bumma, Sarah A. Holstein, Caitlin Costello, Andrzej Jakubowiak, Tanya M. Wildes, Robert Z. Orlowski, Kenneth H. Shain, Andrew J. Cowan, Huiling Pei, Annelore Cortoos, Sharmila Patel, Thomas S. Lin, Peter M. Voorhees, Saad Z. Usmani, Paul G. Richardson

**Affiliations:** 1 Icahn School of Medicine at Mount Sinai, New York, NY USA; 2 Winship Cancer Institute, Emory University, Atlanta, GA USA; 3 Dana-Farber Cancer Institute, Harvard Medical School, Boston, MA USA; 4 Huntsman Cancer Institute, University of Utah School of Medicine, Salt Lake City, UT USA; 5 University of North Carolina–Department of Medicine–Chapel Hill, Chapel Hill, NC USA; 6 Knight Cancer Institute, Oregon Health & Science University, Portland, OR USA; 7 University of Alabama at Birmingham, Birmingham, AL USA; 8 Myeloma, Waldenstrӧm’s and Amyloidosis Program, Simmons Comprehensive Cancer Center, UT Southwestern Medical Center, Dallas, TX USA; 9 Judy and Bernard Briskin Center for Multiple Myeloma Research, City of Hope Comprehensive Cancer Center, Duarte, CA USA; 10 University of California San Francisco, San Francisco, CA USA; 11 Division of Hematology, The Ohio State University Comprehensive Cancer Center, Columbus, OH USA; 12 Division of Oncology and Hematology, Department of Internal Medicine, University of Nebraska Medical Center, Omaha, NE USA; 13 Moores Cancer Center, University of California San Diego, La Jolla, CA USA; 14 University of Chicago Medical Center, Chicago, IL USA; 15 Department of Lymphoma/Myeloma, The University of Texas MD Anderson Cancer Center, Houston, TX USA; 16 Department of Malignant Hematology, H. Lee Moffitt Cancer Center, Tampa, FL USA; 17 Clinical Research Division, Fred Hutch Cancer Center, Seattle, WA USA; 18 Janssen Research & Development, LLC, Titusville, NJ USA; 19 Janssen Scientific Affairs, LLC, Horsham, PA USA; 20 Levine Cancer Institute, Atrium Health Wake Forest University School of Medicine, Charlotte, NC USA; 21 Memorial Sloan Kettering Cancer Center, New York, NY USA

**Keywords:** Targeted therapies, Myeloma

## Abstract

The randomized, phase 2 GRIFFIN study (NCT02874742) evaluated daratumumab plus lenalidomide/bortezomib/dexamethasone (D-RVd) in transplant-eligible newly diagnosed multiple myeloma (NDMM). We present final post hoc analyses (median follow-up, 49.6 months) of clinically relevant subgroups, including patients with high-risk cytogenetic abnormalities (HRCAs) per revised definition (del[17p], t[4;14], t[14;16], t[14;20], and/or gain/amp[1q21]). Patients received 4 induction cycles (D-RVd/RVd), high-dose therapy/transplant, 2 consolidation cycles (D-RVd/RVd), and lenalidomide±daratumumab maintenance (≤ 2 years). Minimal residual disease–negativity (10^−5^) rates were higher for D-RVd versus RVd in patients ≥ 65 years (67.9% vs 17.9%), with HRCAs (54.8% vs 32.4%), and with gain/amp(1q21) (61.8% vs 28.6%). D-RVd showed a trend toward improved progression-free survival versus RVd (hazard ratio [95% confidence interval]) in patients ≥ 65 years (0.29 [0.06–1.48]), with HRCAs (0.38 [0.14–1.01]), and with gain/amp(1q21) (0.42 [0.14–1.27]). In the functional high-risk subgroup (not MRD negative at the end of consolidation), the hazard ratio was 0.82 (0.35–1.89). Among patients ≥ 65 years, grade 3/4 treatment-emergent adverse event (TEAE) rates were higher for D-RVd versus RVd (88.9% vs 77.8%), as were TEAEs leading to discontinuation of ≥ 1 treatment component (37.0% vs 25.9%). One D-RVd patient died due to an unrelated TEAE. These results support the addition of daratumumab to RVd in transplant-eligible patients with high-risk NDMM.

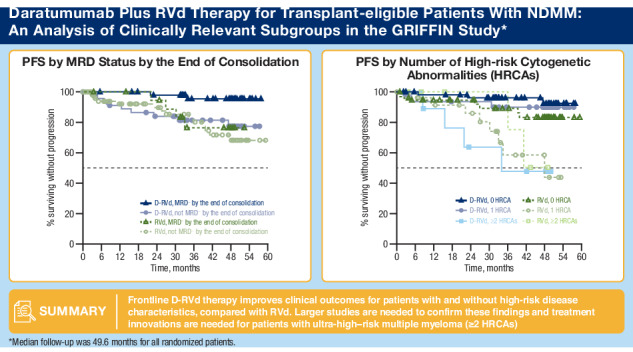

## Introduction

Daratumumab is a human IgGκ monoclonal antibody targeting CD38 with a direct on-tumor [1–4] and immunomodulatory [5–7] mechanism of action, demonstrating greater cytotoxicity toward multiple myeloma (MM) cells ex vivo compared with analogs of other CD38 antibodies [8]. Daratumumab is approved for use in combination with standard-of-care regimens and as a monotherapy for patients with relapsed or refractory MM and in combination with standard-of-care regimens for patients with newly diagnosed MM (NDMM) [9, 10].

The randomized, phase 2 GRIFFIN study (ClinicalTrials.gov Identifier: NCT02874742) evaluated daratumumab in combination with lenalidomide/bortezomib/dexamethasone (D-RVd) in transplant-eligible patients with NDMM [11]. The primary analysis (median follow-up, 13.5 months) showed that D-RVd improved the rate of stringent complete response (sCR) by the end of consolidation versus RVd (42.4% vs 32.0%; 1-sided *P* = 0.068, which met the pre-specified 1-sided α of 0.10) [11]. At the time of final analysis, which occurred after all patients completed ≥ 1 year of long-term follow-up after the end of study treatment, death, or withdrawal (median follow-up, 49.6 months), the rate of complete response or better (≥ CR) was 83.0% in the D-RVd group and 60.2% in the RVd group (*P* = 0.0005). Treatment with D-RVd improved minimal residual disease (MRD)–negativity (10^─5^) rates at the end of maintenance compared to RVd (64.4% vs 30.1%; *P* < 0.0001). This improved depth of response translated into a 55% reduction in the risk of disease progression or death for the D-RVd group versus the RVd group (hazard ratio [HR], 0.45; 95% confidence interval [CI], 0.21–0.95; *P* = 0.0324), and the estimated 48-month progression-free survival (PFS) rates were 87.2% for D-RVd and 70.0% for RVd [12]. These findings are reinforced by recent results from the phase 3 PERSEUS trial investigating the addition of daratumumab to RVd during induction and consolidation and to lenalidomide during maintenance, which demonstrated a nearly identical PFS benefit (HR, 0.42; 95% CI, 0.30–0.59; *P* < 0.001) at a median follow-up of 47.5 months in transplant-eligible patients with NDMM [13].

Although significant improvements have been made in the treatment of MM [14], elderly patients and those with other high-risk features continue to have a poor prognosis [15, 16]. Since effectiveness and duration of response decrease with each subsequent line of therapy, there is a strong rationale to use the most effective treatment regimens up front, particularly for patients with high-risk disease characteristics [17]. As identified by the International Myeloma Working Group (IMWG), the presence of cytogenetic abnormalities influences disease outcomes. Those with high-risk disease can be characterized by the presence of ≥ 1 cytogenetic abnormality, including del(17p), t(4;14), t(14;20), or t(14;16) [18]. Recent advancements in the Revised International Staging System (ISS; ie, R2-ISS) further highlight the high-risk marker gain/amp(1q21) as an important abnormality that plays a prognostic role in MM [19]. Revised high cytogenetic risk can therefore include ≥ 1 of the previously mentioned abnormalities and gain/amp(1q21). Additional high-risk disease characteristics at baseline/time of diagnosis include older age, advanced ISS disease stage, plasma cell leukemia, extramedullary disease, and impaired renal function [20].

Randomized studies are essential to determine the role of novel therapies in high-risk subgroups. Here, we present a post hoc analysis at the time of final analysis (median follow-up overall, 49.6 months) of clinically relevant subgroups from GRIFFIN, including subgroups of patients with high-risk cytogenetic abnormalities (HRCAs) and other baseline high-risk disease characteristics. In addition to these pre-treatment characteristics, suboptimal response to therapy (ie, functional/dynamic/post-treatment high-risk disease) and time to relapse are increasingly recognized as important prognostic variables [21, 22]. Therefore, we also explored the role of daratumumab in functionally high-risk NDMM.

## Methods

### Patients and study design

The full study design of GRIFFIN has been previously reported [11]. Briefly, in the randomized, open-label, phase 2 GRIFFIN study, D-RVd was evaluated in transplant-eligible patients with NDMM. Eligible patients were 18 to 70 years of age, had NDMM as defined by IMWG criteria, had an Eastern Cooperative Oncology Group performance status (ECOG PS) score of ≤ 2, and a creatinine clearance (CrCl) of ≥ 30 mL/min [11]. Patients were randomized 1:1 to receive 4 D-RVd or RVd induction cycles, followed by autologous stem cell transplant (ASCT), then 2 D-RVd or RVd consolidation cycles, and finally up to 2 years of maintenance therapy with D-R or R alone, respectively. After 2 years of study maintenance therapy, patients could continue to receive R therapy per local standard of care [11]. Final analysis occurred after all patients completed ≥ 1 year of long-term follow-up after completion of study maintenance therapy, died, or withdrew. This final analysis included the intent-to-treat population (all randomized patients) and the following patient subgroups: aged ≥ 65 years, ISS stage III disease, high cytogenetic risk per the standard definition (≥ 1 of the following: t[4;14], t[14;16], and/or del[17p]), revised high cytogenetic risk (≥ 1 of the following: t[4;14], t[14;16], del[17p], t[14;20], and/or gain/amp[1q21] [defined as ≥ 3 copies of chromosome 1q21]), 0 HRCA (excluding HRCAs per the revised cytogenetic risk definition), 1 HRCA (per the revised cytogenetic risk definition), ≥ 2 HRCAs (per the revised cytogenetic risk definition), gain/amp(1q21) with or without other HRCAs (per the revised cytogenetic risk definition), gain/amp(1q21) + 1 HRCA (per the revised cytogenetic risk definition), isolated gain/amp(1q21) without other HRCAs, and baseline extramedullary plasmacytomas. Functional risk was also explored and included the following groups of patients: best-confirmed response less than very good partial response (< VGPR) by the end of induction, best-confirmed response of very good partial response or better (≥ VGPR) by the end of induction, MRD negativity by the end of consolidation, not achieving MRD negativity by the end of consolidation, MRD negativity by the end of 2 years of maintenance, and not achieving MRD negativity by the end of 2 years of maintenance.

### Objectives and endpoints

The primary endpoint of GRIFFIN was the sCR rate by the end of consolidation and was previously published [11]. Other endpoints included MRD-negativity rate, overall response rate (ORR), ≥ VGPR rate, ≥ CR rate, sCR rate, PFS, and overall survival [11]. As pre-defined in the study protocol, MRD negativity was measured at a minimum threshold of 10^−^^5^; bone marrow aspirates were collected at baseline, at first evidence of suspected CR or sCR (including patients with ≥ VGPR and suspected daratumumab interference), by the end of induction but before stem cell collection, at the post-ASCT consolidation disease evaluation, and by the end of 1 and 2 years of maintenance therapy. Sustained MRD negativity was evaluated in the intent-to-treat population and was defined as ≥ 2 MRD-negative results in the bone marrow ≥ 12 months apart without any positive result(s) in between. Patients who did not achieve sustained MRD negativity included: those who were MRD positive, those in whom MRD was not determined, those who had disease progression, or those who were MRD negative but subsequently became MRD positive or did not undergo repeat MRD testing (noting MRD testing occurred at intervals as pre-defined in the study protocol). Disease evaluations occurred when CR or sCR was suspected, on the first day of each cycle during induction and consolidation, on Day 21 of Cycle 4 (end of induction), on Day 21 of Cycle 6 (post-ASCT consolidation), every 8 weeks during the maintenance phase, and at the end of study treatment.

### Statistical analysis

Response to study treatment and progressive disease were evaluated using a validated computer algorithm to calculate IMWG response. Rates of best response and MRD negativity were analyzed using the Mantel–Haenszel estimate of the common odds ratio for unstratified tables. PFS was descriptively summarized using the Kaplan–Meier method, and HRs and 95% CIs were obtained from a Cox proportional hazards model with treatment as the sole explanatory variable.

## Results

### Patients

A total of 207 patients were randomized to receive either D-RVd (*n* = 104) or RVd (*n* = 103). Patient baseline demographic and clinical characteristics were previously published [11]. Among randomized patients, each treatment group had similar numbers of patients with high-risk disease characteristics: ≥ 65 years of age (D-RVd *n* = 28, RVd *n* = 28), ISS stage III disease (*n* = 14, *n* = 14), creatinine clearance 30–50 mL/min (*n* = 9, *n* = 9) or > 50 mL/min (*n* = 95, *n* = 94), high cytogenetic risk (del[17p], t[4;14], and/or t[14;16]; *n* = 16, *n* = 14), and revised high cytogenetic risk (with the inclusion of gain/amp[1q21] and/or t[14;20]; *n* = 42, *n* = 37).

Patients were further divided into cytogenetic risk groups by HRCA according to the revised high-risk definition (presence of t[4;14], t[14;16], del[17p], t[14;20], and/or gain/amp[1q21]), noting it was not possible to distinguish gain versus amplification and therefore outcomes were evaluated among patients with ≥ 3 copies of 1q21. For this analysis, the following groups were evaluated: 0 HRCA (D-RVd *n* = 56, RVd *n* = 60), 1 HRCA (*n* = 32, *n* = 29), ≥ 2 HRCAs (*n* = 10, *n* = 8), gain/amp(1q21) (*n* = 34, *n* = 28), gain/amp(1q21) plus 1 other HRCA (*n* = 9, *n* = 6), or gain/amp(1q21) without other HRCAs (*n* = 25, *n* = 22). There were a few patients with extramedullary plasmacytomas (D-RVd *n* = 1, RVd *n* = 2) whose outcomes were explored but not reported due to small patient numbers.

### Efficacy

**Fig. 1:**
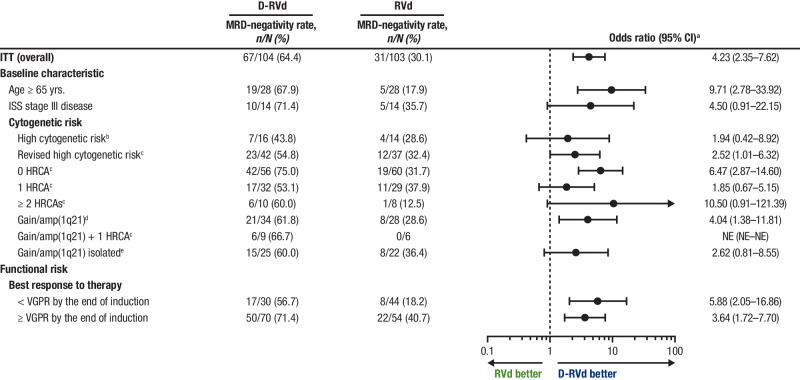
Subgroup analysis of MRD-negativity (10^−^^5^) rates by the end of the study. MRD-negativity rates for all groups were evaluated at the time of the final analysis (median overall follow-up, 49.6 months). MRD was evaluated by next-generation sequencing using the clonoSEQ assay (v2.0; Adaptive Biotechnologies, Seattle, WA) at a minimum sensitivity threshold of 1 in 100,000 cells (10^−^^5^) in alignment with IMWG criteria [44]. MRD minimal residual disease, D-RVd daratumumab plus lenalidomide/bortezomib/dexamethasone, RVd lenalidomide/bortezomib/dexamethasone, CI confidence interval, ITT intent-to-treat, ISS International Staging System, HRCA high-risk cytogenetic abnormality, NE not evaluable, VGPR very good partial response, FISH fluorescence in situ hybridization. ^a^Mantel–Haenszel estimate of the common odds ratio for unstratified tables is used. An odds ratio > 1 indicates an advantage for D-RVd. ^b^High-risk cytogenetics are defined based on FISH testing as ≥ 1 of the following: del(17p), t(4;14), or t(14;16). ^c^Revised high-risk cytogenetics are defined based on FISH testing as ≥ 1 HRCA: del(17p), t(4;14), t(14;16), t(14;20), or gain/amp(1q21) (≥ 3 copies of chromosome 1q21). ^d^Patients in this group have gain/amp(1q21) with or without other HRCAs (del[17p], t[4;14], t[14;16], or t[14;20]). ^e^Patients with isolated gain/amp(1q21) do not have any other HRCAs.

**Fig. 2:**
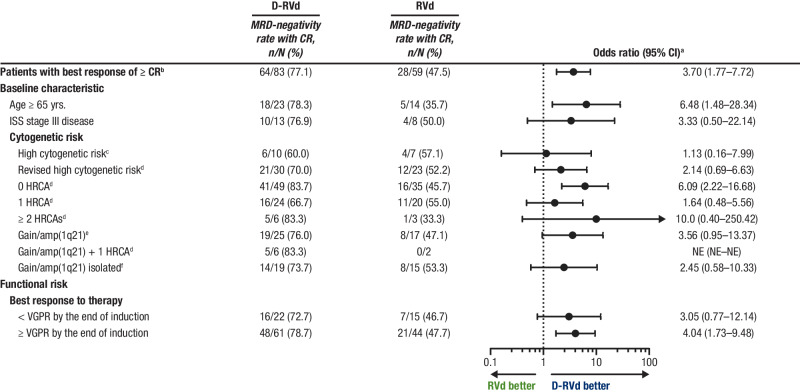
Subgroup analysis of MRD-negativity (10^−^^5^) rates among patients with a best response of ≥ CR by the end of the study. MRD-negativity rates were evaluated among response-evaluable patients^a^ who achieved a best response of ≥ CR and were measured at the time of the final analysis (median follow-up in overall population, 49.6 months). MRD was evaluated by next-generation sequencing using the clonoSEQ assay (v2.0; Adaptive Biotechnologies, Seattle, WA) at a minimum sensitivity threshold of 1 in 100,000 cells (10^−^^5^) in alignment with IMWG criteria [44]. MRD minimal residual disease, D-RVd daratumumab plus lenalidomide/bortezomib/dexamethasone, RVd lenalidomide/bortezomib/dexamethasone, CI confidence interval, ITT intent-to-treat, ISS International Staging System, HRCA high-risk cytogenetic abnormality, NE not evaluable, VGPR very good partial response, FISH fluorescence in situ hybridization. ^a^Mantel–Haenszel estimate of the common odds ratio for unstratified tables is used. An odds ratio > 1 indicates an advantage for D-RVd. ^b^This analysis included patients from the response-evaluable population, which included all randomized patients who had measurable disease (confirmed MM diagnosis), received ≥ 1 dose of study treatment, and had ≥ 1 postbaseline disease assessment. ^c^High-risk cytogenetics are defined based on FISH testing as ≥ 1 of the following: del(17p), t(4;14), or t(14;16). ^d^Revised high-risk cytogenetics are defined based on FISH testing as ≥ 1 HRCA: del(17p), t(4;14), t(14;16), t(14;20), or gain/amp(1q21) (≥ 3 copies of chromosome 1q21). ^e^Patients in this group have gain/amp(1q21) with or without other HRCAs (del[17p], t[4;14], t[14;16], or t[14;20]). ^f^Patients with isolated gain/amp(1q21) do not have any other HRCAs.

**Fig. 3:**
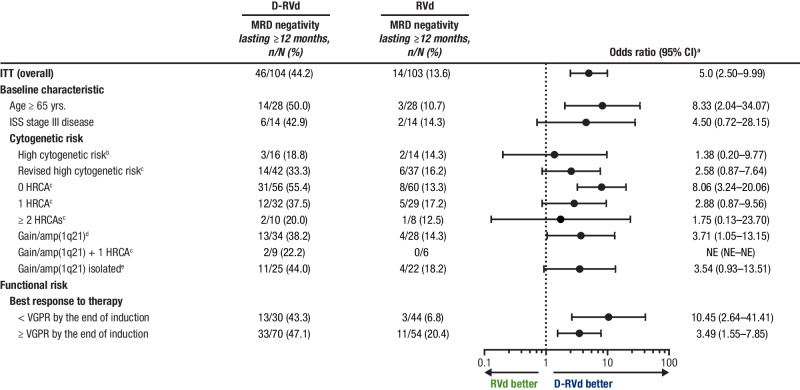
Subgroup analysis of rates of sustained MRD negativity (10^−^^5^) lasting ≥ 12 months. MRD-negativity rates for all groups were evaluated at the time of the final analysis (median overall follow-up, 49.6 months). MRD was evaluated by next-generation sequencing using the clonoSEQ assay (v2.0; Adaptive Biotechnologies, Seattle, WA) at a minimum sensitivity threshold of 1 in 100,000 cells (10^−^^5^) in alignment with IMWG criteria [44]. MRD minimal residual disease, D-RVd daratumumab plus lenalidomide/bortezomib/dexamethasone, RVd lenalidomide/bortezomib/dexamethasone, CI confidence interval, ITT intent-to-treat, ISS International Staging System, HRCA high-risk cytogenetic abnormality, NE not evaluable, VGPR very good partial response, FISH fluorescence in situ hybridization. ^a^Mantel–Haenszel estimate of the common odds ratio for unstratified tables is used. An odds ratio > 1 indicates an advantage for D-RVd. ^b^High-risk cytogenetics are defined based on FISH testing as ≥ 1 of the following: del(17p), t(4;14), or t(14;16). ^c^Revised high-risk cytogenetics are defined based on FISH testing as ≥ 1 HRCA: del(17p), t(4;14), t(14;16), t(14;20), or gain/amp(1q21) (≥ 3 copies of chromosome 1q21). ^d^Patients in this group have gain/amp(1q21) with or without other HRCAs (del[17p], t[4;14], t[14;16], or t[14;20]). ^e^Patients with isolated gain/amp(1q21) do not have any other HRCAs.

At the time of final analysis, MRD-negativity (10^−^^5^) rates were higher for D-RVd versus RVd in all patient subgroups, including patients with ultra-high–risk disease (≥ 2 HRCAs) and functionally high-risk patients (defined as a best response of < VGPR by the end of induction; Fig. 1). In an analysis of response by the end of the study, sCR rates were higher for D-RVd versus RVd for most subgroups (Supplemental Fig. 1). Among patients who achieved a best response of ≥ CR by the end of the study, MRD-negativity (10^−^^5^) rates were higher for D-RVd versus RVd in all patient subgroups (Fig. 2). The subgroup size was small for patients with high cytogenetic risk disease (defined as ≥ 1 of the following: del17p, t(4;14), or t(14;16); D-RVd *n* = 10, RVd *n* = 7), and caution should be used when interpreting findings in this subgroup. Consistent with the trend of higher MRD-negativity rates for D-RVd across subgroups, D-RVd was also associated with higher rates of sustained MRD negativity (10^−5^) lasting ≥ 12 months in all subgroups (Fig. 3). No patient who achieved sustained MRD negativity (10^−^^5^) lasting ≥ 12 months developed progressive disease. Among MRD-evaluable patients who achieved MRD negativity (10^−^^5^) at any time, 2 D-RVd patients (both with ≥ 2 HRCAs) and 5 RVd patients (3 with 1 HRCA and 2 with 0 HRCA) developed progressive disease. Both D-RVd patients and 3 RVd patients developed progressive disease after initially becoming MRD positive again. The remaining 2 RVd patients developed progressive disease while they were still considered MRD negative but did not have MRD samples collected around the time of disease progression.

**Fig. 4:**
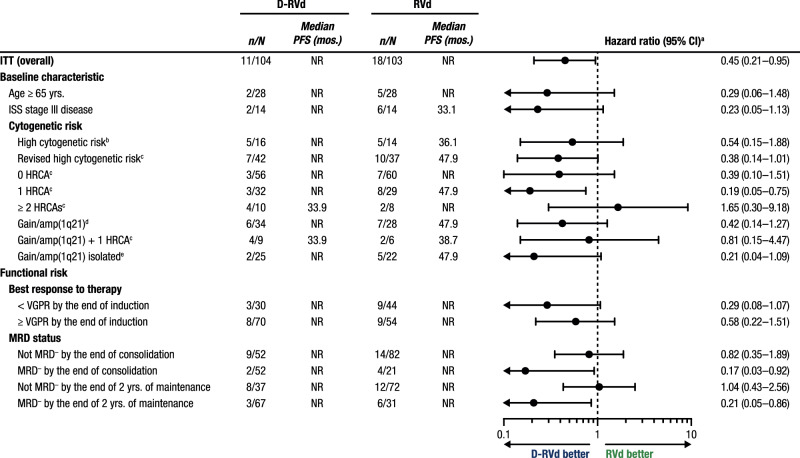
Subgroup analysis of PFS. Results of the PFS HR point estimates and their 95% CIs among clinically relevant subgroups of patients. PFS analyses for all groups were evaluated at the time of the final analysis (median overall follow-up, 49.6 months). PFS progression-free survival, D-RVd daratumumab plus lenalidomide/bortezomib/dexamethasone, RVd lenalidomide/bortezomib/dexamethasone, CI confidence interval, ITT intent-to-treat, NR not reached, ISS International Staging System, NE not evaluable, HRCA high-risk cytogenetic abnormality, VGPR very good partial response, MRD minimal residual disease, FISH fluorescence in situ hybridization, HR hazard ratio. ^a^HR and 95% CI are from a Cox proportional hazards model with treatment as the sole explanatory variable. An HR < 1 indicates an advantage for D-RVd. ^b^High-risk cytogenetics are defined based on FISH testing as ≥ 1 of the following: del(17p), t(4;14), or t(14;16). ^c^Revised high-risk cytogenetics are defined based on FISH testing as ≥ 1 HRCA: del(17p), t(4;14), t(14;16), t(14;20), or gain/amp(1q21) (≥ 3 copies of chromosome 1q21). ^d^Patients in this group have gain/amp(1q21) with or without other HRCAs (del[17p], t[4;14], t[14;16], or t[14;20]). ^e^Patients with isolated gain/amp(1q21) do not have any other HRCAs.

**Fig. 5:**
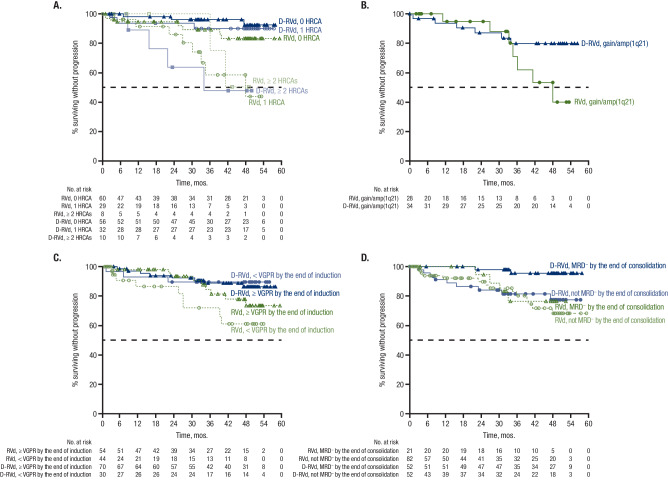
Subgroup analysis of PFS. PFS is shown **A** by NDMM disease with 0, 1, or ≥ 2 HRCAs^a^, **B** among patients with gain/amp(1q21)^b^, **C** by VGPR status by the end of induction, and **D** by MRD status (10^−^^5^) by the end of consolidation. Results of the Kaplan–Meier estimates of PFS among clinically relevant subgroups of patients are shown and were evaluated at the time of the final analysis (median follow-up, 49.6 months). PFS progression-free survival, VGPR very good partial response, MRD minimal residual disease, NDMM newly diagnosed multiple myeloma, HRCA high-risk cytogenetic abnormality, D-RVd daratumumab plus lenalidomide/bortezomib/dexamethasone, RVd lenalidomide/bortezomib/dexamethasone, FISH fluorescence in situ hybridization. ^a^HRCA groups are based on FISH testing as the absence (0 HRCA) or presence of ≥ 1 of the following: del(17p), t(4;14), t(14;16), t(14;20), or gain/amp(1q21) (≥ 3 copies of chromosome 1q21). ^b^Patients in this group have gain/amp(1q21) with or without other HRCAs (del[17p], t[4;14], t[14;16], or t[14;20]).

At 49.6 months of overall median follow-up, HR point estimates for PFS among subgroups with cytogenetic abnormalities indicate a trend toward improvement with D-RVd versus RVd, except among patients with ≥ 2 HRCAs (Fig. 4). Among patients with 0 HRCA, median PFS was not reached for either treatment group, and the PFS HR was 0.39 (95% CI, 0.10–1.51) for D-RVd versus RVd (Fig. 4 and Fig. 5A). For patients with 1 HRCA, median PFS was not reached for D-RVd and was 47.9 months for RVd, and the PFS HR was 0.19 (95% CI, 0.05–0.75) for D-RVd versus RVd (Fig. 4 and Fig. 5A). In patients with ≥ 2 HRCAs, median PFS was 33.9 months for D-RVd and was not reached for RVd (HR, 1.65; 95% CI, 0.30–9.18; Fig. 4 and Fig. 5A); however, results should be interpreted with caution due to the small sample size (D-RVd *n* = 10, RVd *n* = 8). Among patients with gain/amp(1q21) with or without other HRCAs, PFS was not reached for D-RVd and was 47.9 months for RVd, and the PFS HR was 0.42 (95% CI, 0.14–1.27) for D-RVd versus RVd (Fig. 4 and Fig. 5B).

In a PFS analysis by functional risk, median PFS was not reached regardless of VGPR status by the end of induction, and HR point estimates for PFS were consistently lower than 1, indicating a trend toward improvement with D-RVd versus RVd (≥ VGPR: HR, 0.58; 95% CI, 0.22–1.51; < VGPR: HR, 0.29; 95% CI, 0.08–1.07; Fig. 5C). Also, within the functionally high-risk subgroup of patients with a best response of < VGPR by the end of induction, more D-RVd patients had revised high cytogenetic risk at baseline compared with RVd (D-RVd 48.3% [*n* = 14/29], RVd 32.6% [*n* = 14/43]) and 1 HRCA (37.9% [*n* = 11/29], 23.3% [*n* = 10/43]), and similar proportions had ≥ 2 HRCAs (10.3% [*n* = 3/29], 9.3% [*n* = 4/43]; Supplementary Table 1). Additionally, when PFS was evaluated by achievement of MRD negativity (10^−^^5^), median PFS was not reached in either treatment group regardless of MRD status by the end of consolidation (Fig. 5D). HR point estimates for PFS were lower than 1, indicating a trend toward improvement with D-RVd versus RVd among patients who were MRD negative by the end of consolidation (HR, 0.17; 95% CI, 0.03–0.92) and for those who did not achieve MRD negativity by the end of consolidation (HR, 0.82; 95% CI, 0.35–1.89; Fig. 4). Similar to what was seen among patients with best response < VGPR by the end of induction, the proportion of patients with revised high cytogenetic risk at baseline was higher for D-RVd versus RVd among patients who did not achieve MRD negativity by the end of consolidation (D-RVd 52.1% [*n* = 25/48], RVd 34.2% [*n* = 26/76]), as was the proportion of patients with 1 HRCA (39.6% [*n* = 19/48], 25.0% [*n* = 19/76]). Also in this functionally high-risk subgroup, a slightly higher proportion of D-RVd patients had ≥2 HRCAs (D-RVd 12.5% [*n* = 6/48], RVd 9.2% [*n* = 7/76]; Supplementary Table 1). Among patients who did not achieve MRD negativity by the end of maintenance, there was no PFS benefit for D-RVd versus RVd therapy (Fig. 4).

### Safety

Table 1 provides a summary of the most common (> 30%) treatment-emergent adverse events (TEAEs) in the safety analysis population (all randomized patients who received ≥ 1 dose of study treatment) separated by age < 65 years (D-RVd *n* = 72, RVd *n* = 75) and ≥ 65 years (*n* = 27, *n* = 27). The 3 most common TEAEs of any grade for patients aged < 65 years and ≥ 65 years were fatigue (< 65 years: D-RVd 66.7%, RVd 60.0%; ≥ 65 years: D-RVd 85.2%, RVd 66.7%), diarrhea (66.7%, 52.0%; 66.7%, 63.0%), and peripheral neuropathy (56.9%, 74.7%; 77.8%, 81.5%). The rates of grade 3/4 TEAEs were slightly higher for D-RVd versus RVd both among patients aged < 65 years (D-RVd 84.7%, RVd 80.0%) and patients aged ≥ 65 years (88.9%, 77.8%). Grade 3/4 TEAEs occurring in > 20% of patients included neutropenia, which had a higher rate for D-RVd in both age subgroups (< 65 years: D-RVd 50.0%, RVd 20.0%; ≥ 65 years: D-RVd 37.0%, RVd 29.6%), and lymphopenia, which occurred in a similar proportion of patients aged < 65 years across treatment groups (D-RVd 22.2%, RVd 26.7%), and in more D-RVd patients aged ≥ 65 years (25.9%, 11.1%). Among patients aged < 65 years, the incidence of serious TEAEs was lower in the D-RVd group (D-RVd 41.7%, RVd 56.0%); however, for patients aged ≥ 65 years, the D-RVd group had more serious events (59.3%, 40.7%). For patients aged < 65 years, the most common serious TEAEs were pneumonia (D-RVd 12.5%, RVd 18.7%) and pyrexia (12.5%, 13.3%). For patients aged ≥ 65 years, the most common serious TEAE in the D-RVd group was pneumonia (D-RVd 22.2%, RVd 0%), and the most common in the RVd group was pulmonary embolism (0%, 7.4%). TEAEs that led to the discontinuation of ≥ 1 therapeutic agent were comparable between those aged < 65 years (D-RVd 31.9%, RVd 33.3%) and higher for D-RVd among those aged ≥ 65 years (37.0%, 25.9%), with peripheral neuropathy being the most common TEAE leading to discontinuation of ≥ 1 drug in both subgroups (< 65 years: D-RVd 11.1%, RVd 13.3%; ≥ 65 years: D-RVd 18.5%, RVd 11.1%). Among patients aged < 65 and ≥ 65 years, the incidence of TEAEs leading to lenalidomide dose reduction was higher for the D-RVd group (< 65 years: D-RVd 33.3%, RVd 28.0%; ≥ 65 years: D-RVd 59.3%, RVd 33.3%), with neutropenia being the most common TEAE leading to lenalidomide dose reduction for both age subgroups (15.3%, 5.3%; 22.2%, 14.8%). Death as an outcome of a TEAE occurred in 1 patient aged < 65 years (RVd *n* = 1 [death, cause unknown]) and 1 patient aged ≥ 65 years (D-RVd *n* = 1 [pneumonia]). Both deaths were considered unrelated to study treatment by the treating investigator.

**Table 1:** Most common (> 30%)^a^ any grade TEAEs by age (< 65 years and ≥ 65 years).

	< 65 years		≥ 65 years	
Most common TEAEs, *n* (%)	D-RVd (*n* = 72)	RVd (*n* = 75)	D-RVd (*n* = 27)	RVd (*n* = 27)
Hematologic				
Neutropenia	47 (65.3)	29 (38.7)	16 (59.3)	12 (44.4)
Thrombocytopenia	30 (41.7)	24 (32.0)	14 (51.9)	12 (44.4)
Leukopenia	29 (40.3)	21 (28.0)	10 (37.0)	9 (33.3)
Anemia	25 (34.7)	25 (33.3)	12 (44.4)	8 (29.6)
Lymphopenia	23 (31.9)	23 (30.7)	8 (29.6)	6 (22.2)
Nonhematologic				
Upper respiratory tract infection	51 (70.8)	37 (49.3)	16 (59.3)	14 (51.9)
Diarrhea	48 (66.7)	39 (52.0)	18 (66.7)	17 (63.0)
Fatigue	48 (66.7)	45 (60.0)	23 (85.2)	18 (66.7)
Peripheral neuropathy^b^	41 (56.9)	56 (74.7)	21 (77.8)	22 (81.5)
Nausea	38 (52.8)	37 (49.3)	14 (51.9)	14 (51.9)
Constipation	37 (51.4)	29 (38.7)	14 (51.9)	13 (48.1)
Insomnia	36 (50.0)	25 (33.3)	9 (33.3)	6 (22.2)
Cough	35 (48.6)	26 (34.7)	18 (66.7)	5 (18.5)
Pyrexia	34 (47.2)	27 (36.0)	14 (51.9)	6 (22.2)
Back pain	30 (41.7)	29 (38.7)	11 (40.7)	7 (25.9)
Arthralgia	27 (37.5)	26 (34.7)	12 (44.4)	12 (44.4)
Headache	27 (37.5)	18 (24.0)	6 (22.2)	6 (22.2)
Muscle spasms	26 (36.1)	11 (14.7)	4 (14.8)	9 (33.3)
Vomiting	25 (34.7)	21 (28.0)	7 (25.9)	8 (29.6)
Peripheral edema	24 (33.3)	25 (33.3)	12 (44.4)	12 (44.4)
Hypokalemia	19 (26.4)	20 (26.7)	9 (33.3)	7 (25.9)
Pain in extremity	19 (26.4)	13 (17.3)	3 (11.1)	9 (33.3)
Dyspnea	14 (19.4)	24 (32.0)	10 (37.0)	7 (25.9)
Dizziness	15 (20.8)	16 (21.3)	8 (29.6)	9 (33.3)
Pneumonia	14 (19.4)	16 (21.3)	10 (37.0)	2 (7.4)
Dysgeusia	14 (19.4)	14 (18.7)	9 (33.3)	5 (18.5)

*TEAE* treatment-emergent adverse event, *D-RVd* daratumumab plus lenalidomide/bortezomib/dexamethasone, *RVd* lenalidomide/bortezomib/dexamethasone.

^a^Includes TEAEs occurring in ≥ 30% of patients aged < 65 years or ≥ 65 years in either treatment group from the safety analysis population (all randomized patients who received ≥ 1 dose of study treatment).

^b^Includes preferred terms neuropathy peripheral and peripheral sensory neuropathy.

## Discussion

Although novel therapies have greatly improved outcomes for patients with NDMM, certain subgroups of patients experience suboptimal long-term outcomes, including those with older age, advanced ISS disease stage, and high-risk cytogenetic abnormalities [23–26]. Here, we describe results from a post hoc analysis of clinically relevant subgroups from the phase 2 GRIFFIN study in which transplant-eligible patients with NDMM received D-RVd or RVd induction/consolidation, ASCT, and D-R or R maintenance. In all subgroups evaluated, D-RVd was associated with higher MRD-negativity (10^−^^5^) rates than RVd. Given the lack of difference between regimens in subgroups following analysis of sCR and the broad differences observed with MRD, these data support the use of MRD negativity as a more reliable indicator of response outcomes. In addition, the PFS HR point estimates were less than 1, indicating a trend toward improvement with D-RVd versus RVd in the majority of subgroups, with a notable HR of 0.17 (95% CI, 0.03–0.92) achieved by those who were MRD negative by the end of consolidation. This trend was not observed, however, in patients with ultra-high–risk disease, defined as ≥ 2 HRCAs, or those who did not achieve MRD negativity by the end of maintenance.

Patients with MM with high-risk chromosomal abnormalities have a worse prognosis compared to patients with no or standard-risk cytogenetic abnormalities. Moreover, the prognosis varies depending on the number and type of genetic features, as well as therapy choice [18]. In the phase 3 DETERMINATION trial, the use of early ASCT with RVd induction therapy resulted in superior PFS versus RVd alone, with a greater improvement observed in patients with high-risk compared to standard-risk disease [27]. Additionally, while response rates were similar between those receiving RVd versus RVd + ASCT, high-risk patients achieved much higher ≥ CR rates with early ASCT versus standard therapy [28], emphasizing the importance of tailored treatment based on high-risk cytogenetics. With recent advances in MM therapies, there is focused research to determine if outcomes among high-risk patients can be improved by the use of novel agents, particularly in the frontline setting. Phase 3 clinical trials of daratumumab plus other standard-of-care regimens in transplant-eligible patients with NDMM (CASSIOPEIA) and transplant-ineligible NDMM (ALCYONE and MAIA) demonstrated that treatment with daratumumab improves PFS for patients with high-risk cytogenetics compared to standard-of-care regimens [29–31]. High-risk cytogenetics were defined as ≥ 1 of the following at baseline for ALCYONE and MAIA: del(17p), t(4;14), and t(14;16); and for CASSIOPEIA: del(17p) and t(4;14). Further validation of the outcomes of these individual clinical trials came from meta-analyses and pooled analyses of patient-level data from MAIA, ALCYONE, and/or CASSIOPEIA, which also showed that the addition of daratumumab to backbone therapy reduced the risk of disease progression or death by 23% to 41% compared to backbone therapy alone among patients with high-risk cytogenetics [32–34]. Furthermore, recent results from the phase 3 PERSEUS trial also demonstrated a PFS benefit with D-RVd versus RVd in transplant-eligible patients with NDMM across clinically relevant subgroups, including patients with high-risk cytogenetics, defined as the presence of del(17p), t(4;14), and/or t(14;16) (HR, 0.59; 95% CI, 0.36–0.99) [13]. In this final post hoc subgroup analysis of GRIFFIN, D-RVd showed a trend toward improvement of PFS for patients with high cytogenetic risk features according to the standard high-risk definition (≥ 1 of the following: del[17p], t[4;14], t[14;16]), according to a revised high-risk definition (also including t[14;20] and/or gain/amp[1q21]), and among patients with 1 HRCA (according to the revised definition) compared to RVd therapy. For patients with ultra-high–risk disease (≥ 2 HRCAs), no benefit was observed. The results in ultra-high–risk disease should be interpreted cautiously due to the small sample size but suggest novel treatment approaches are needed for this group of patients.

A high-risk feature of interest includes the gain or amplification of 1q21, which is among the most common of high-risk chromosomal abnormalities observed in patients with MM at diagnosis, occurring with a frequency of 30% to 40% [35]. Gain(1q21) and amp(1q21) can be distinguished by the presence of 3 copies or ≥ 4 copies [35], respectively; however, in GRIFFIN, due to the way that cytogenetic data were collected, it was not possible to distinguish gain(1q21) versus amp(1q21); therefore, outcomes were evaluated among patients with ≥ 3 copies of 1q21 (gain/amp[1q21]), similar to the MASTER trial [36]. Numerous studies have shown that the gain/amp(1q21) is associated with poor prognosis among patients with MM; specifically in transplant-eligible patients with NDMM, gain/amp(1q21) is associated with impaired PFS and overall survival compared to patients without gain/amp(1q21) [35]. Reports on the anti-CD38 antibody isatuximab showed that isatuximab-based therapies (isatuximab plus pomalidomide and dexamethasone, or isatuximab plus carfilzomib and dexamethasone) improved PFS for patients with relapsed or refractory MM and gain/amp(1q21) versus standard-of-care therapy alone [37, 38]. In our present analysis of GRIFFIN, in which 32.7% (34/104) of D-RVd patients and 27.2% (28/103) of RVd patients had gain/amp(1q21), the addition of daratumumab to RVd seems to be associated with favorable outcomes among patients with high-risk features, including patients with gain/amp(1q21). D-RVd achieved higher rates of MRD negativity, rates of MRD negativity with ≥ CR, rates of durable MRD negativity lasting ≥ 12 months, and PFS compared with RVd among patients with gain/amp(1q21) with or without other HRCAs, gain/amp(1q21) with 1 HRCA, and gain/amp(1q21) without any other HRCAs. Together, our data suggest frontline D-RVd therapy may provide clinical benefit to patients with gain/amp(1q21) versus RVd therapy, although larger studies like the PERSEUS study are needed to confirm this initial observation.

This post hoc analysis of GRIFFIN also evaluated other subgroups associated with poor prognosis, including ISS stage III disease, age ≥ 65 years, and those with functional high-risk disease (suboptimal responders, defined in this analysis as patients who failed to achieve ≥ VGPR by the end of induction or who did not achieve MRD negativity by the end of consolidation). In all subgroups evaluated, D-RVd was associated with higher MRD-negativity (10^−^^5^) rates than RVd. PFS HR point estimates were less than 1, thus indicating a trend toward improvement with D-RVd in all subgroups except those with ≥ 2 HRCAs or functionally high-risk patients who did not achieve MRD negativity after 2 years of maintenance therapy. Functionally high-risk patients, by definition, have suboptimal responses after induction or consolidation. A higher number of D-RVd patients with suboptimal response had baseline revised high cytogenetic risk compared to RVd patients; however, it is important to note that this needs to be interpreted in regard to the total number of patients. The overall number of functionally high-risk patients was lower in the D-RVd group compared to the RVd group, but the absolute number of patients with cytogenetic abnormalities was the same in both treatment groups (Supplementary Table 1), thus leading to an enrichment of patients with high cytogenetic risk in the functionally high-risk D-RVd group. However, the overall proportions of patients with 0, 1, and ≥ 2 HRCAs were generally comparable to proportions among all randomized patients (Supplementary Table 1). This aligns with prior reports showing that many patients experience disease progression even in the absence of common high-risk cytogenetic abnormalities and underscores the need to expand current risk stratification and treatment approaches for these patients [21, 22]. Despite the benefit of daratumumab-containing regimens, future studies are warranted for high-risk subgroups to investigate additional novel therapeutic strategies. These may include the use of BCMA-targeting CAR-T cells and bispecific antibodies, which have shown fast, deep responses in patients with refractory MM [23], as well as CAR-T and bispecific antibodies targeting against other antigens, such as GPRC5D.

In an analysis of safety among patients aged < 65 years and ≥ 65 years in GRIFFIN, rates of grade 3/4 TEAEs were slightly higher for D-RVd versus RVd in both age subgroups. TEAEs leading to discontinuation of ≥ 1 study treatment component were similar for D-RVd versus RVd among patients aged < 65 years; however, among patients aged ≥ 65 years, rates were higher in the D-RVd group. Overall, 1 patient receiving D-RVd (aged ≥ 65 years) and 1 patient receiving RVd (aged < 65 years) died due to a TEAE, both of which were considered unrelated to study treatment by the investigator. Previous post hoc analyses of the MAIA study [39] and ALCYONE study [40] in transplant-ineligible patients with NDMM showed that frailer patients generally had higher rates of grade 3/4 TEAEs, serious TEAEs, and TEAEs leading to treatment discontinuation versus other frailty subgroups [39, 40]; nevertheless, clinically meaningful improvements and preservation of health-related quality of life and Global Health Status were seen [41, 42]. While these studies are not directly comparable, both studies showed that daratumumab-based therapies provide clinical benefit among older patients with preservation of quality of life despite the increase in incidence of adverse events. Of note, patient-reported outcomes data from GRIFFIN suggest D-RVd resulted in greater improvements in quality of life combined with longer disease management [43].

This post hoc analysis included several limitations. First, for most subgroups, sample sizes were relatively small, limiting the robustness of the observed data, which are descriptive in nature, and, consequently, the definitiveness of our conclusions. Additionally, while MRD serves as an indicator for response outcomes, there are associated limitations, such as identifying the optimal timepoint(s) for MRD assessment. Another limitation is the multiple testing completed in this post hoc analysis (multiple subgroups across multiple timepoints), as the study was not designed for such comparisons. Thus, larger phase 3 clinical trials with greater sample sizes of patients in high-risk categories are needed to draw more definitive conclusions. Furthermore, due to the disproportionate dropout rate seen between study arms (high dropout rate for suboptimal responses with RVd vs D-RVd), there may have been bias against the experimental arm. The lack of data available on lactate dehydrogenase prevented the use of the Revised ISS as a further subgroup of interest. Lastly, due to the way cytogenetic data were collected in GRIFFIN, we had limited information available on the clonal burden for 17p or chromosome 1q21 and therefore could not distinguish gain versus amplification of 1q21. However, data on outcomes among patients with ≥ 3 copies of 1q21 (gain/amp[1q21]) were still valuable given its association with impaired PFS and overall survival [35].

In summary, this post hoc analysis of GRIFFIN shows that the use of daratumumab-based quadruplet therapy in transplant-eligible patients with NDMM with high cytogenetic risk and other poor prognostic characteristics showed a trend toward the improvement of clinical outcomes versus standard-of-care triplet therapy. However, ultra-high–risk patients with ≥ 2 HRCAs continue to do poorly. Two large ongoing phase 3 trials, CEPHEUS (D-VRd vs VRd in NDMM without intent to transplant) and PERSEUS (D-VRd vs VRd in transplant-eligible NDMM) will provide further insight and confirm these findings on the value of daratumumab-based quadruplet regimens in ultra-high−risk patients. However, continued research is needed for patients with ≥ 2 HRCAs to explore treatment innovations beyond prolonged consolidation and maintenance therapy.

### Supplementary information

Supplemental Information

## Data Availability

The data sharing policy of Janssen Pharmaceutical Companies of Johnson & Johnson is available at https://www.janssen.com/clinical-trials/transparency. As noted on this site, requests for access to the study data can be submitted through Yale Open Data Access (YODA) Project site at http://yoda.yale.edu.
