# Supplementary material for: Daratumumab in transplant-eligible patients with newly diagnosed multiple myeloma: final analysis of clinically relevant subgroups in GRIFFIN
Source: Blood Cancer J. 2024 Jul 8;14(1):107. doi: 10.1038/s41408-024-01088-6 (PMC11231363; doi:10.1038/s41408-024-01088-6)

# SUPPLEMENTARY INFORMATION

**Daratumumab in transplant-eligible patients with newly diagnosed multiple myeloma: final analysis of clinically relevant subgroups in GRIFFIN**

Ajai Chari, Jonathan L. Kaufman, Jacob Laubach, Douglas W. Sborov, Brandi Reeves, Cesar Rodriguez, Rebecca Silbermann, Luciano J. Costa, Larry D. Anderson Jr, Nitya Nathwani, Nina Shah, Naresh Bumma, Sarah A. Holstein, Caitlin Costello, Andrzej Jakubowiak, Tanya M. Wildes, Robert Z. Orlowski, Kenneth H. Shain, Andrew J. Cowan, Huiling Pei, Annelore Cortoos, Sharmila Patel, Thomas S. Lin, Peter M. Voorhees, Saad Z. Usmani, Paul G. Richardson

# Supplementary Table 1. Summary of baseline cytogenetic risk according to the revised definition^a^ in the overall population of randomized patients and among those with functional high risk (suboptimal response to therapy including patients with <VGPR by the end of induction or those who did not achieve MRD negativity by the end of consolidation)

|  | **D-RVd** | **RVd** |
| --- | --- | --- |
| **ITT** |  |  |
| Evaluable patients | n = 98 | n = 97 |
| Standard risk (0 HRCAs) | 56 (57.1) | 60 (61.9) |
| Revised high risk | 42 (42.9) | 37 (38.1) |
| 1 HRCA | 32 (32.7) | 29 (29.9) |
| ≥2 HRCAs | 10 (10.2) | 8 (8.2) |
| **Patients with <VGPR by the end of induction** |  |  |
| Evaluable patients | n = 29 | n = 43 |
| Standard risk (0 HRCAs) | 15 (51.7) | 29 (67.4) |
| Revised high risk | 14 (48.3) | 14 (32.6) |
| 1 HRCA | 11 (37.9) | 10 (23.3) |
| ≥2 HRCAs | 3 (10.3) | 4 (9.3) |
| **Patients who did not achieve MRD negativity (10^−5^) by the end of consolidation** |  |  |
| Evaluable patients | n = 48 | n = 76 |
| Standard risk (0 HRCAs) | 23 (47.9) | 50 (65.8) |
| Revised high risk | 25 (52.1) | 26 (34.2) |
| 1 HRCA | 19 (39.6) | 19 (25.0) |
| ≥2 HRCAs | 6 (12.5) | 7 (9.2) |

Abbreviations: D-RVd, daratumumab plus lenalidomide/bortezomib/dexamethasone; RVd, lenalidomide/bortezomib/dexamethasone; ITT, intent to treat; HRCA, high-risk cytogenetic abnormality; VGPR, very good partial response; MRD, minimal residual disease; FISH, fluorescence in situ hybridization.

^a^Revised high-risk cytogenetics are defined based on FISH testing (≥1 of the following: t[4;14], t[14;16], del[17p], t[14;20], and/or gain/amp[1q21] [≥3 copies of chromosome 1q21]). Revised standard risk is defined as the absence of HRCAs per the revised cytogenetic risk definition.

# Supplementary Figure 1. Subgroup analysis of sCR by the end of study.

Results of sCR and their 95% CIs among the overall response evaluable population^a^ and clinically relevant subgroups of patients measured at the time of the final analysis (median follow-up in overall population, 49.6 months). sCR, stringent complete response; D-RVd, daratumumab plus lenalidomide/bortezomib/dexamethasone; RVd, lenalidomide/bortezomib/dexamethasone; CI, confidence interval; ITT, intent-to-treat; ISS, International Staging System; HRCA, high-risk cytogenetic abnormality; VGPR, very good partial response; MRD, minimal residual disease; FISH, fluorescence in situ hybridization.

^a^Mantel–Haenszel estimate of the common odds ratio for unstratified tables is used. An odds ratio >1 indicates an advantage for D-RVd .

^b^This analysis included patients from the response evaluable population, which included all randomized patients who had measurable disease (confirmed MM diagnosis), received ≥1 dose of study treatment, and had ≥1 postbaseline disease assessment.

^c^High-risk cytogenetics are defined based on FISH testing as ≥1 of the following: del(17p), t(4;14), or t(14;16).

^d^Revised high-risk cytogenetics are defined based on FISH testing as ≥1 HRCA: del(17p), t(4;14), t(14;16), t(14;20), or gain/amp(1q21) (≥3 copies of chromosome 1q21).

^e^Patients in this group have gain/amp(1q21) with or without other HRCAs (del[17p], t[4;14], t[14;16], or t[14;20]).

^f^Patients with isolated gain/amp(1q21) do not have any other HRCAs.


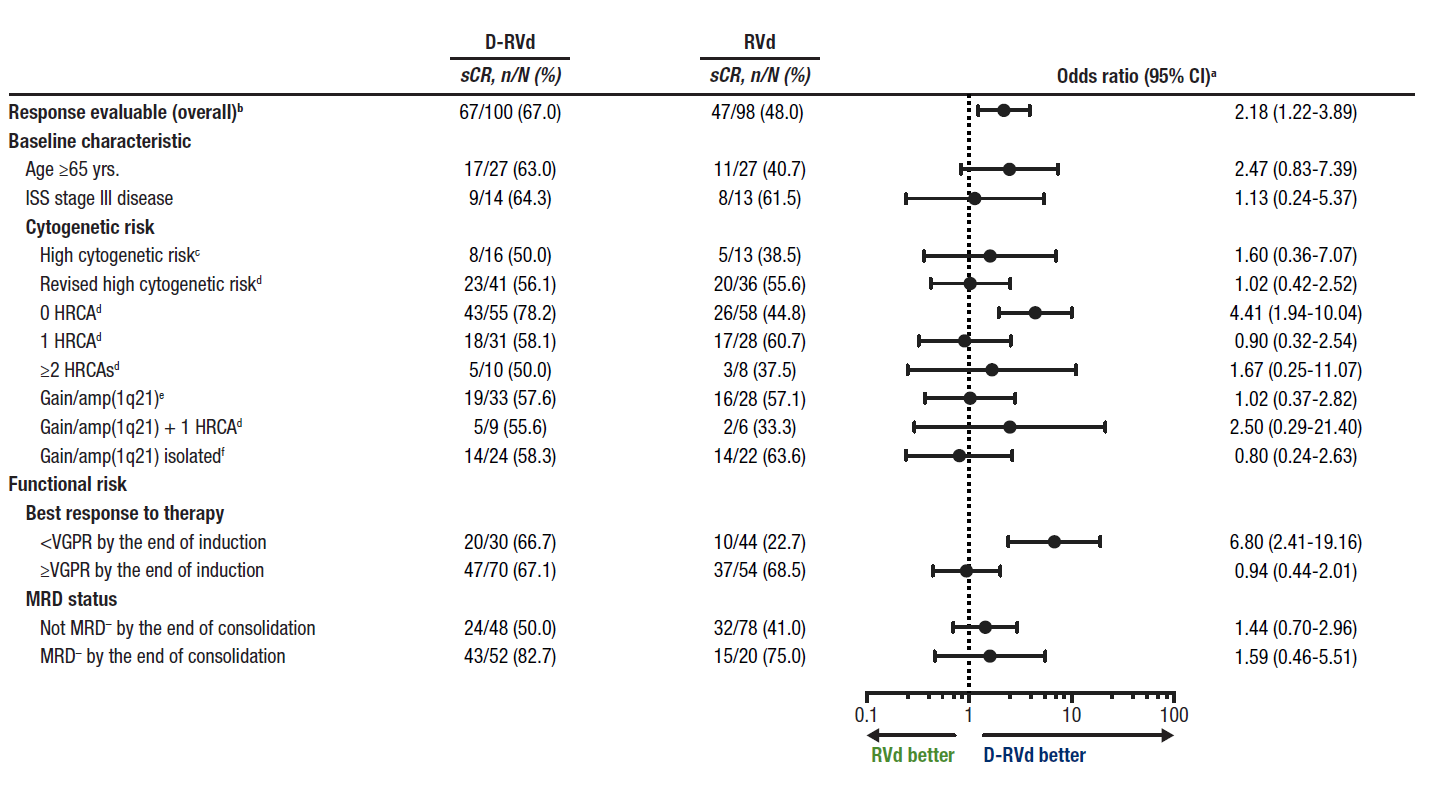

Supplement: Supplementary file 2 — Supplemental Information [file 41408_2024_1088_MOESM1_ESM.docx]
